# Comparison and Suitability of Gel Matrix for Entrapping Higher Content of Enzymes for Commercial Applications

**DOI:** 10.4103/0250-474X.65010

**Published:** 2010

**Authors:** R. Mahajan, V. K Gupta, J. Sharma

**Affiliations:** Department of Biotechnology, Kurukshetra University, Kurukshetra-136 119, India; 1 Department of Biochemistry, Kurukshetra University, Kurukshetra-136 119, India

**Keywords:** Agar/agarose gel, calcium alginate, entrapment, enzyme, immobilization, polyacrylamide

## Abstract

To check the suitability of enzyme entrapped beads for use in pharmaceutical industry, amylase enzyme was entrapped in agar/agarose, polyacrylamide gels and calcium alginate beads. Sodium alginate of 1% concentration was found to be best with respect to immobilization efficiency and calcium alginate beads so obtained were not much susceptible to breakage. When sodium alginate- amylase mixture was added from a height of about 20-30 cm. into CaCl_2_ solution, size of beads was large at higher alginate concentration due to the increase in the size of droplet formation before entering into CaCl_2_ solution. Enzyme entrapped polyacrylamide and agar/agarose gels were fragile and could not withstand repeated use whereas enzyme entrapped in large calcium alginate beads was used successfully for 50 cycles for the conversion of starch into product without much damage to the beads under stirring conditions. Amylase preparation was also mixed with urease, lysozyme and coimmobilized in large sized calcium alginate beads. These beads were used for 10 repeated cycles to check the conversion of substrates into their products by their respective enzymes and we concluded that an enzyme or mixture of two or three enzymes can be immobilized in the same large sized calcium alginate beads. This will save the additional cost of bioreactor, manpower, maintenance conditions required for the conversion of one drug into another using enzyme/s entrapped in large sized beads.

Knowledge of immobilization of enzymes has led to exploit more fully the potential of enzymes for industrial purposes. The cost of producing the enzymes is often high so immobilized enzyme preparations may be more effective, since they are recoverable and can be used repeatedly in bioreactor or for other purposes, remain separated during the reaction by physical entrapment or other methods and more stable than free enzymes. Many instances of successful application of immobilized cells/ enzymes from bacterial, fungal, higher plant, mammalian cells and subcellular organelles in/onto different matrices by different immobilization methods have been reported[1–11].

Various methods used for immobilization of enzymes are adsorption, covalent binding, entrapment and membrane confinement. The cost of immobilization by covalent binding and membrane confinement is high as compared to entrapment methods. Various types of gels are used for entrapment of enzymes. The enzyme may be entrapped within polymeric mesh such as agar, polyacrylamide gel or calcium alginate by carrying out the polymerization reaction and /or cross – linking reaction in the presence of enzyme[12–14].

Polyacrylamide, the most commonly used matrix for the entrapment of enzymes, has the property of being non- ionic. A number of procedures for the entrapment of enzymes in polyacrylamide gels have been published[15,16]. Trevan and Grover described the procedure for the immobilization of urease[15]. Thus one may have to modify the buffers and pH values some what to meet the specific requirement of enzymes.

Agar is a complex polysaccharide consisting of 3,6-anhydro-L-galactose and D-galacto pyranose, free of nitrogen, produced from various red algae belonging to *Gelidium, Gracilaria, Gigartino* and *pterocladia*. It liquifies on heating to 96° and hardens into a jelly on cooling to 40-45°. Agar/ agarose have been used for the entrapment of cells in the form of spherical beads, blocks and membranes. Matsunaga *et al*, have described entrapment of *Clostridium butyricum* in agar[17].

Alginate, the major structural polysaccharide of marine brown algae, contains β-D-mannopyranosyl uronate and α-L-gulopyranosyl uronate in regular (1-4)-linked sequences. Both homopolymeric sequences are found together, although to different extents, in all alginate molecules. In the presence of monovalent cations, the polysaccharide forms a viscous solution even at high concentrations, whereas in the presence of divalent cations, especially Ca^[2+]^, gelation occurs. Since gel formation can take place under mild conditions, entrapment in this matrix is very suitable for immobilization of enzymes, drugs, cells, proteins[18–25]. Increasing the concentration of alginate solution will result in more tightly cross-linked gels[26].

Immobilization of enzyme by entrapment method can be done by very simple procedure and moreover, the cost of immobilization is low as compared to other methods. Our main aim of investigation was to find out the gel matrix suitable for immobilization of enzymes using various entrapment methods and to check the viability of enzyme entrapped beads for use in pharmaceutical industry.

## MATERIALS AND METHODS

Agar, agarose, bisacrylamide were purchased from Himedia laboratories Ltd. Mumbai, India. Sodium alginate and acrylamide were from Loba Chemie, Mumbai, India. CaCl_2_ (fused) was purchased from Ranbaxy Laboratories Ltd. India. Tetramethylethylenediamine (TEMED) was from Sigma, St. Louis, USA. All other chemicals used for the assay of enzymes were of analytical grade. Standard lysozyme and *Micrococcus luteus* were from Banglore Genei, Banglore, India. Amylase enzyme was used for all the entrapment methods. Same batch of amylase was used for comparison of entrapment in different gels.

Amylase enzyme was immobilized by gel entrapment method in calcium alginate, polyacrylamide, agar/agarose gels. Agar/agarose (0.25, 0.5, 1.0, 1.5 and 2.0%), polyacrylamide (7.5, 10.0, 12.5, 15 and 20%), sodium alginate (0.25, 0.5, 1.0, 1.5 and 2.0 %) and CaCl_2_ (0.1, 0.2, 0.3, 0.4, 0.5 and 1.0 M) were used for entrapment of enzyme and entrapped enzyme was checked for the conversion of substrate into product up to 50 repeated cycles. A mixture of enzymes (amylase, urease and lysozyme) was entrapped in the same calcium alginate beads and checked for the ability of these enzymes to convert their respective substrates into their products.

### Entrapment in agar/agarose gel:

The solution of agar/agarose of concentrations 0.5, 1, 2, 3 and 4% were heated separately to liquify the agar. Gel was allowed to cool to 45-50° and then equal amount of amylase in phosphate buffer (25 ml) was added to it so that the final concentration of agar was 0.25, 0.5, 1, 1.5 and 2%, respectively. The agar-amylase mixture was then allowed to solidify onto the glass plate and cut into small blocks and used for the conversion of starch into maltose for 50 repeated cycles.

### Entrapment in polyacrylamide gel:

Polyacrylamide gel is the most commonly used material for entrapment. Method given by Freeman and Aharonowitz for the entrapment of cells by polyacrylamide gel was modified[16]. For the preparation of 15% gel, 7.5 g acrylamide, 0.5 g bisacrylamide, 50 mg ammonium persulfate was added to 25 ml of phosphate buffer, pH 6.8 and mixed to dissolve these solids. Then 25 ml of amylase solution added. Mixed properly and added 50 µl of TEMED. Mixed gently and poured into glass Petri dishes, or gel casting vertical electrophoresis unit in order to get the gel of uniform and desired thickness (with thick gels, problem was of diffusion of substrate). Polymerization was done at room temperature for 1 h. The gel was cut into small pieces and suspended in 0.1 M phosphate buffer till further use. Similarly for the preparation of other concentrations of gel, 3.75, 5.0, 6.25 and 10.0 g of acrylamide was added to get the final concentration of 7.5, 10.0, 12.5, and 20%.

### Entrapment in calcium alginate gel:

For calcium alginate beads, 25 ml of 0.5, 1, 2, 3 and 4% solutions of sodium alginate were prepared and mixed with equal volume of amylase solution to get the final concentration of sodium alginate 0.25, 0.5, 1, 1.5 and 2%, respectively. Entrapment of enzyme in calcium alginate gel was done by modifying the method of Kierstan and Bucke[21]. Different CaCl_2_ concentrations (0.1, 0.2, 0.3, 0.4 and 0.5 M) were used to optimize the best concentration. Different concentrations of sodium alginate (0.5, 1, 2, 3 and 4%) mixed with enzyme solution were added separately from a height of nearly 1-2 cm and 20-30 cm, into excess of CaCl_2_ solution. For 50 ml sodium alginate- enzyme mixture, 500 ml of CaCl_2_ solution was used.

All these gels were kept at 4° after proper washings till further use. For the estimation of enzyme activity, the gels were dried in the air. The beads prepared by entrapment of enzyme amylase in polyacrylamide gel, agar/agarose and calcium alginate were checked for the conversion of starch into maltose for 50 repeated cycles. Overhead stirrer was finally used for mixing the reaction mixture instead of magnetic stirrer.

A mixture of enzymes (amylase, urease and lysozyme) was entrapped in calcium alginate beads and checked the ability of these enzymes to convert their respective substrates into their products. Urease was taken from jackbean meal and lysozyme from egg white. Amylase, urease and lysozyme enzymes were filtered separately, concentrated by ultrafiltration and then phosphate buffer of pH 7.0 was added to this preparation to give final concentration of 0.1 M.

### Enzyme activity:

Amylase activity was measured by digesting 5 ml of 1% starch solution to achromic point. The time required for the conversion of starch into maltose was noted by change in colour of iodine solution from blue to colourless[27]. Time of conversion of starch into maltose was noted for entrapped enzyme in agar, polyacrylamide and calcium alginate gels and also for free enzyme as control. For the estimation of lysozyme activity, *Micrococcus luteus* was used to check the lysis by lysozyme. The activity was calculated using the standard lysozyme with known activity. Absorbance of *Micrococcus luteus* solution was taken at 450 nm before and after the addition of lysozyme to check the lysis[27]. Urease enzyme activity was estimated by the liberation of ammonia from urea by the enzyme. The ammonia is reacted with Nessler's reagent to form yellow colouration which is measured at 480 nm[28].

## RESULTS AND DISCUSSION

In case of agar/agarose, polyacrylamide and sodium alginate, the different concentrations were tried to choose the best concentration of gel with respect to immobilization efficiency as well as working efficiency of the beads with respect to diffusion of substrate into the beads (Tables 1 and 2). Agar at concentration of 1.5% was found to be optimum with respect to working efficiency. For calcium alginate beads, the concentration of CaCl_2_ was also checked with respect to immobilization efficiency and 0.5 M concentration of CaCl_2_ was found to be optimum. Different concentrations of sodium alginate showed difference in the diffusion of substrate into the beads and with increase in the concentration of sodium alginate, diffusion ability of the substrate into the beads decreased. This is due to more cross-linking at higher concentrations with more calcium binding sites that is why beads at high concentration appear bright white in colour[26]. At 0.5% concentration of sodium alginate or below, immobilization efficiency was high but these gels can not withstand wear tear and were very fragile. Sodium alginate of 1% was found to be best with respect to immobilization efficiency and diffusion of substrate into the beads. Moreover, this gel can withstand breakage. So the results indicate that high concentrations of alginates are difficult to work with. In case of polyacrylamide gel, immobilization and working efficiency was also checked and 15% concentration was found to be best with respect to working efficiency. At lower concentrations, gels were fragile and enzyme was loosely bound to the gel. Immobilization of enzymes, drugs, cells, proteins has been reported by several workers but one has to modify the conditions depending on the material to be immobilized and application of immobilized preparation[18–25].

**TABLE 1:** COMPARISON OF IMMOBILISATION EFFICIENCY AND WORKING EFFICIENCY (%) OF BEADS

Gel concentration (%)	Immobilization effi ciency (%) on the basis of amylase activity in the supernatant		Working effi ciency (%) of beads on the basis of enzyme activity	
	Agarose gel	Calcium alginate	Agarose gel	Calcium alginate
0.25	45-50	93-95	33	85
0.50	65-70	98-99	59	90
1.0	88-92	98-99	78	93
1.5	88-93	98-99	88	86
2.0	93-95	98-99	78	81

Enzyme activity was taken after washing with 0.1 M phosphate buffer (pH 7.0) thrice to remove loosely bound enzyme. These values are mean of six observations

**TABLE 2:** COMPARISON OF IMMOBILISATION EFFICIENCY AND WORKING EFFICIENCY (%) OF BEADS

Gel concentration (%)	Immobilization effi ciency (%) on the basis of amylase activity in the supernatant	Working effi ciency (%) of beads on the basis of enzyme activity
	Polyacrylamide gel	Polyacrylamide gel
7.5	78-82	59
10.0	88-92	67
12.5	92-95	79
15.0	93-95	89
20.0	97-99	75

Enzyme activity was taken after washing with 0.1 M phosphate buffer (pH 7.0) thrice to remove loosely bound enzyme. These values are mean of six observations

**Fig. 1:**
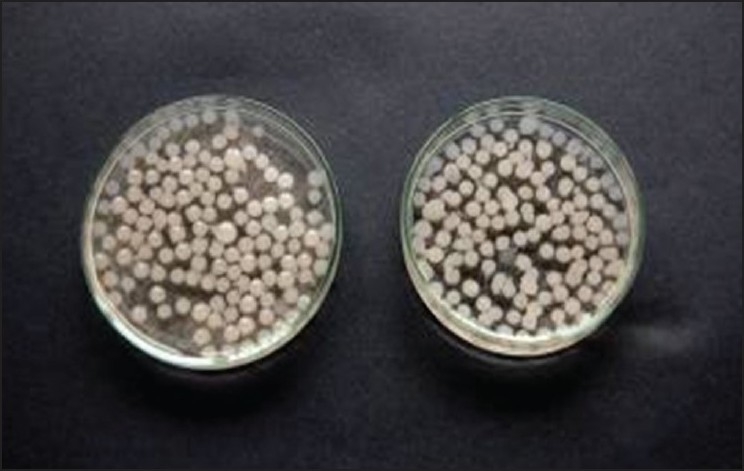
Calcium alginate beads formed from 1-2 cm. These beads were obtained after sodium alginate addition from a height of 1-2 cm in to the CaCl_2_ solution.

**Fig. 2:**
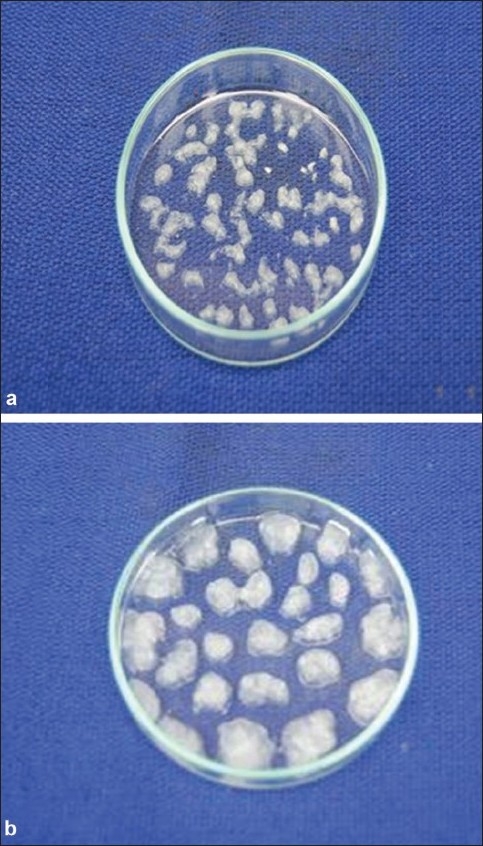
Calcium alginate beads formed from 20-30 cm These beads were obtained after adding sodium alginate from a height of 20-30 cm in to the CaCl_2_ solution (a) sodium alginate concentration 0.25 % (b) sodium alginate concentration 1%.

When sodium alginate-amylase mixture was added just from 1-2 cm height above into the CaCl_2_ solution, all beads (at concentrations 1, 1.5 and 2%) were spherical in shape (fig. 1) and at lower concentrations (below 1%) their shape was not perfectly spherical and were mainly elliptical, ovoid in shape. The spherical shape of beads at higher sodium alginate concentrations is due to high viscosity at higher concentrations whereas at lower concentrations due to low viscosity of the solution, drop falls immediately into the CaCl_2_ solution as compared to higher concentrations of sodium alginate. Beads at 1.5 and 2% concentrations of sodium alginate were bright white in colour and at low concentrations (below 1%), they were light white and transparent in colour. The beads at all concentrations were small and nearly of same size. When the sodium alginate-amylase preparation was added from the height of 20-30 cm into the CaCl_2_ solution, size of beads was larger at higher concentration (fig. 2) and decreased with the decrease in the concentration of sodium alginate. The beads were not spherical at any concentration because they fall into the solution from a height. Large sized beads at higher concentration are due to the big size droplets formed from a height, due to more viscosity at higher concentration. The entrapment of enzyme per bead basis was more in large sized beads as compared to small sized beads formed at lower concentrations of sodium alginate and also the amount of enzyme entrapped (per gram of alginate basis) was high in large sized beads. So this indicates that large sized beads will save the additional cost required for small sized beads to accommodate the same amount of enzyme in terms of bioreactor size.

Beads when tried for the conversion of starch into maltose for 50 cycles, beads having sodium alginate concentration 0.5% and below were fragile, showed decrease in the activity after every cycle. The beads at higher concentrations (1.5 and 2%) were resistant to breakage but the main problem was of diffusion of substrate (Table 3). Sodium alginate of 1% concentration was found to be optimum with respect to working efficiency of calcium alginate beads.

**TABLE 3:** COMPARISON OF WORKING EFFICIENCY OF THE CALCIUM ALGINATE BEADS

Sodium alginate concentration (%)	Enzyme activity (%)				
	10 cycles	20 cycles	30 cycles	40 cycles	50 cycles
0.25	85	76	62	50	33
0.50	91	84	74	64	51
1.0	95	90	85	80	75
1.5	85	79	73	68	61
2.0	80	72	66	61	55

Initial activity of enzyme was taken as 100%. Working effi ciency of the calcium alginate beads was calculated on the basis of enzyme activity at different concentrations of sodium alginate

In order to check the immobilization efficiency of different gels, amylase enzyme entrapped gels were washed three times with 0.1 M phosphate buffer, pH 7.0 and immobilization efficiency was calculated on the basis of activity of enzyme in the supernatant. Although the efficiency was highest in calcium alginate beads but the conversion of substrate into product decreased at higher concentrations of sodium alginate. This is due to the decrease in the diffusion of the substrate into the beads.

Entrapped enzyme in polyacrylamide gel, agar/ agarose gel took nearly 16-19 min to convert starch into maltose as compared to the free enzyme, where the time required for conversion was nearly 11 min. In case of calcium alginate entrapped enzyme, the time required to convert starch was nearly 13-15 min. This indicates that the time for the conversion of starch into maltose has increased in case of entrapped enzyme as compared to free enzyme and further the conversion time in case of calcium alginate entrapped enzyme was less as compared to enzyme in polyacrylamide, agar/agarose gel.

The different gels entrapping amylase enzyme were used to check their susceptibility to breakage by stirring with overhead stirrer and magnetic stirrer for 10 repeated cycles. Stirring of the reaction mixture with overhead stirrer caused less breakage of the beads as compared to stirring with magnetic stirrer. The activity of amylase enzyme entrapped in polyacrylamide, agar/agarose gel and calcium alginate beads left after 10 repeated cycles was 56, 44, 95% and 29, 08, 86% in the presence of overhead and magnetic stirring, respectively. The gels of polyacrylamide and agar were fragile and can not be used for repeated conversion of substrate into product. The gels of polyacrylamide and agar have been used for immobilizing enzymes[15–17].

Amylase enzyme immobilized in different gels was checked for 50 repeated cycles for the conversion of starch into maltose. Amylase enzyme activity left after 25 cycles in polyacrylamide, agar/agarose gel and calcium alginate beads was 18, 12 and 88%, respectively. The activity of amylase enzyme in calcium alginate beads after 50 cycles was 78% where as in polyacrylamide and agar/agarose gels, activity was less than 5% after 30 cycles. In case of polyacrylamide/agar/agarose gels, the activity of amylase was decreased, as these gels were fragile. So 1% concentration of sodium alginate under all conditions was found to be suitable with respect to immobilization efficiency and conversion of substrate into product.

When all the enzyme entrapped gels were packed in separate columns and checked for the conversion efficiency, it was again highest in calcium alginate beads as compared to polyacrylamide gel and agar/agarose gels.

In order to check the effect of these matrices on the entrapped enzyme, the activity of the amylase enzyme entrapped in all these gel matrices was checked by keeping these gels at 4° and 25° and compared with the free enzyme control, also kept at 4° and 25°. In all these matrices, the decrease in activity pattern was nearly same when compared with the control. This indicates that these matrices have no adverse effect on the activity of the enzyme.

Amylase enzyme preparation was then mixed with urease and lysozyme and the mixture was immobilized in 1% sodium alginate and 0.5 M CaCl_2_. The large sized beads so obtained were used for 10 repeated cycles to check the conversion of substrates into their products by their respective enzymes (Table 4). The activity of all the three enzymes was in the range of 95 to 97% after 10 repeated cycles. So we concluded that the Immobilized enzyme in large sized calcium alginate gel works well than in agar/agarose and polyacrylamide gels. In our study, we have used three different enzymes producing three separate products. Therefore, three products can be obtained simultaneously in one reactor using the immobilized enzymes in large-sized beads. A mixture of two or three enzymes of series of reaction may be immobilized by entrapment method in a big sized calcium alginate beads to get directly the end product of series, but the reaction conditions, pH etc. should be taken into consideration. This will save the additional cost of bioreactor, manpower, maintenance conditions required for the drug conversions, as the reactions can take place in one step.

**TABLE 4:** ACTIVITY OF VARIOUS ENZYMES ENTRAPPED IN THE SAME CALCIUM ALGINATE GEL

Enzyme	Activity after 5 cycles	Activity after 10 cycles
Urease	98	97
Lysozyme	98	95
Amylase	97	95

Initial activity of enzymes was taken as 100%.
